# The Relationship between Religiosity and Cognitive Function among Chinese Older Adults in Chicago

**DOI:** 10.1093/geroni/igab046.2636

**Published:** 2021-12-17

**Authors:** Jacob Koppel Egierd, Stephanie Bergren, Lisa Lanza, Dana Dytchwald, XinQi Dong

**Affiliations:** 1 Rutgers University New, Monroe Township, New Jersey, United States; 2 Rutgers University, New Brunswick, New Jersey, United States; 3 Rutgers University, Rutgers Institute for Health, New Jersey, United States

## Abstract

Evidence suggests religiosity may be related to cognitive decline in older adults living in the US and China. However, the relationship between religiosity and cognitive function has not been tested in a Chinese community in the US. Immigration and isolation often cause diasporas to differ from communities where they currently reside and their origin. This study aims to determine the relationship between religiosity, cognitive function, and demographic attributes in a sample of older Chinese adults age 60 to 105 living in the Chicago area (N = 3157). Regression analysis showed participation in organized religion significantly predicted higher global cognitive function (β = 0.031, p < 0.001, N = 3051). Of all cognitive function measures including episodic memory (East Boston Memory Immediate and Delayed Recall Test), perceptual speed (Symbol Digit Modalities Test), working memory (Digit Backwards Test), cognitive impairment (Mini Mental State Examination), and a composite measure of (global cognition), the importance of religion only significantly predicted greater working memory capacity (β = 0.045, p = 0.003, N = 3058). Practicing religion at home had a nonsignificant relationship with all measures of cognitive function. All analyses controlled for the following covariates: gender, education, income, number of children, marital status, and health insurance coverage status. Findings suggest that among aspects of religiosity, organized religious involvement may have a positive association with higher cognitive function. Future research should explore between-population differences in the relationships of social factors, religiosity, and cognition function to determine what practices can best benefit older adults in various communities.

